# Implementation of coordinated global serotype 2 oral poliovirus vaccine cessation: risks of potential non-synchronous cessation

**DOI:** 10.1186/s12879-016-1536-9

**Published:** 2016-05-26

**Authors:** Radboud J. Duintjer Tebbens, Lee M. Hampton, Kimberly M. Thompson

**Affiliations:** Kid Risk, Inc., 10524 Moss Park Rd., Ste. 204-364, Orlando, FL 32832 USA; Global Immunization Division, Center for Global Health, Centers for Disease Control and Prevention, Atlanta, GA USA

**Keywords:** Polio, Eradication, Risk management, Oral poliovirus vaccine, Dynamic modeling, Vaccine-derived poliovirus

## Abstract

**Background:**

The endgame for polio eradication involves coordinated global cessation of oral poliovirus vaccine (OPV) with cessation of serotype 2 OPV (OPV2 cessation) implemented in late April and early May 2016 and cessation of serotypes 1 and 3 OPV (OPV13 cessation) currently planned for after 2018. The logistics associated with globally switching all use of trivalent OPV (tOPV) to bivalent OPV (bOPV) represent a significant undertaking, which may cause some complications, including delays that lead to different timing of the switch across shared borders.

**Methods:**

Building on an integrated global model for long-term poliovirus risk management, we consider the expected vulnerability of different populations to transmission of OPV2-related polioviruses as a function of time following the switch. We explore the relationship between the net reproduction number (R_n_) of OPV2 at the time of the switch and the time until OPV2-related viruses imported from countries still using OPV2 can establish transmission. We also analyze some specific situations modeled after populations at high potential risk of circulating serotype 2 vaccine-derived poliovirus (cVDPV2) outbreaks in the event of a non-synchronous switch.

**Results:**

Well-implemented tOPV immunization activities prior to the tOPV to bOPV switch (i.e., tOPV intensification sufficient to prevent the creation of indigenous cVDPV2 outbreaks) lead to sufficient population immunity to transmission to cause die-out of any imported OPV2-related viruses for over 6 months after the switch in all populations in the global model. Higher R_n_ of OPV2 at the time of the switch reduces the time until imported OPV2-related viruses can establish transmission and increases the time during which indigenous OPV2-related viruses circulate. Modeling specific connected populations suggests a relatively low vulnerability to importations of OPV2-related viruses that could establish transmission in the context of a non-synchronous switch from tOPV to bOPV, unless the gap between switch times becomes very long (>6 months) or a high risk of indigenous cVDPV2s already exists in the importing and/or the exporting population.

**Conclusions:**

Short national discrepancies in the timing of the tOPV to bOPV switch will likely not significantly increase cVDPV2 risks due to the insurance provided by tOPV intensification efforts, although the goal to coordinate national switches within the globally agreed April 17-May 1, 2016 time window minimized the risks associated with cross-border importations.

## Background

The polio endgame includes the coordinated global cessation of use of oral poliovirus vaccine (OPV), with the cessation of use of serotype 2 OPV (OPV2) currently planned for April 17-May 1, 2016. The cessation of use of OPV2 will take the form of the synchronized replacement of trivalent OPV (tOPV), which contains attenuated poliovirus serotypes 1, 2, and 3, with bivalent OPV (bOPV), which contains only attenuated poliovirus serotypes 1 and 3 [1, 2]. A successful switch from tOPV to bOPV (the switch) will help pave the way for the coordinated global cessation of use of OPV serotypes 1 and 3 (OPV13 cessation) following the global certification of the eradication of serotypes 1 and 3 wild poliovirus (WPV). The attenuated polioviruses in OPV mutate when they replicate and over time can develop into circulating vaccine-derived polioviruses (cVDPVs) that behave like wild polioviruses (WPVs) with respect to transmissibility and their ability to cause paralysis. Although ending the use of a given OPV serotype will end the introduction of new OPV viruses of the serotype that could evolve to cVDPVs, some risk exists of cVDPV outbreaks after OPV cessation due to continued propagation and evolution of OPV-related viruses of the serotype already present in the population as population immunity to transmission with that poliovirus serotype declines [3]. Current efforts to prevent serotype 2 cVDPV (cVDPV2) cases from occurring after the switch include increased use of tOPV in supplemental immunization activities (SIAs) in the run up to the switch to increase population immunity to serotype 2 transmission (i.e., tOPV intensification [4, 5]), preparedness for continued surveillance and outbreak response in the event of detection of OPV2-related virus circulation after the switch, [6, 7] introduction of inactivated poliovirus vaccine (IPV) into routine immunization (RI) programs, and plans for tight synchronization of the switch within and between countries [8].

Previous modeling provided insights about the importance of efforts to prevent cVDPV2 cases after the switch. An integrated global model for long-term poliovirus risk management (i.e., the global model) [4] suggests that well-implemented tOPV intensification will prevent creation of indigenous cVDPV2s after a globally-coordinated switch in April 2016. The global model also indicates that failure to implement tOPV intensification (e.g., through continued reliance on bOPV for most SIAs in high-risk populations prior to the switch) will lead to cVDPV2 outbreaks after the switch. If cVDPV outbreaks of any serotype occur, aggressive outbreak response with monovalent OPV (mOPV) can potentially control any virus re-introductions that might occur during the first 5 years after OPV cessation of that serotype in developing countries, although mOPV use for outbreak response beyond approximately 5 years after homotypic OPV cessation comes with challenges because it may create new risks [4, 7]. Fortunately, the risk of poliovirus reintroductions occurring 5 or more years after complete cessation of OPV (e.g., containment failures, immunodeficiency-associated vaccine-derived polioviruses (iVDPVs)) should primarily affect relatively higher-income countries that can control outbreaks with IPV [4, 7]. Consequently, a global analysis assuming well-implemented tOPV intensification, well-coordinated OPV2 and OPV13 cessation, and aggressive outbreak response with mOPV (while available and allowable) or IPV (long-term) suggested a low risk of uncontrolled outbreaks for a strategy of OPV cessation followed by 5 years of global IPV use. Such a policy would lead to expected incremental net benefits during 2013–2052 of approximately $15 billion (2013 net present value and 2013 US dollars) compared to continued OPV use through 2052 [4]. Thus, it is possible to plan and implement tOPV intensification for most or all places that need SIAs to boost population immunity to serotype 2 polioviruses and establish a sufficiently large mOPV stockpile to enable aggressive outbreak response if needed using mOPV [7]. However, questions remain regarding the logistics and effectiveness of implementing the planned tightly-synchronized, globally-coordinated switch from tOPV to bOPV involving 156 OPV-using countries, including some countries affected by civil disorder, natural disasters, and/or other disruptions [8].

The rapidly approaching switch and subsequent OPV13 cessation represent huge global operations that require unprecedented coordination of immunization programs between and within countries. For example, the need to intensify tOPV use in SIAs in high-risk areas at the same time that vaccine manufacturers prepare to stop tOPV production in anticipation of the switch requires careful management of vaccine supplies. Underestimation of tOPV needs or misallocation of tOPV could create a tOPV shortage in some countries, [5] which in turn could lead those countries to stop using tOPV before the planned global switch in April 2016. For example, if global health leaders had decided to postpone the switch globally due to insufficient confidence in the interruption of persistent cVDPV2s [9] (e.g., in the event of detection of large numbers of cVDPV2 cases in the months leading up to April 2016), tOPV supply challenges could have become even more severe and could have resulted in gaps in switch dates between countries and/or challenges to sufficient tOPV intensification prior to the revised global switch date. Even with sufficient tOPV supply, some risk exists that not all countries or areas within countries can or will effectively switch at the same time. As of April 2016, insufficient global supplies of IPV present challenges for countries in the run up to the switch and the situation led to some consideration of delaying national switch dates. Any gaps in switch times represent a concern because population immunity to transmission will markedly drop fairly rapidly after OPV2 cessation in most places [3, 10].

IPV use will prevent paralysis in successfully vaccinated recipients, but provides only limited intestinal immunity, as indicated by clinical trials [10–12] and the circulation of serotype 1 WPV (WPV1) in Israel without cases of paralytic polio during 2013–2014 despite high IPV coverage [13, 14]. The ability of IPV to prevent transmission depends on the intensity of fecal-oral transmission. In places with moderate to low fecal-oral transmission, IPV-only may provide sufficient population immunity to transmission to prevent evolution of less transmissible viruses (e.g., closely related to OPV) to evolve to cVDPVs, which may explain why Israel detected widespread WPV1 transmission without widespread cVDPV transmission despite likely importation of OPV viruses from bordering countries that used OPV [15]. However, in areas at highest risk of cVDPV2 outbreaks after the switch, characterized by low RI coverage and intense fecal-oral transmission, IPV use will likely not significantly increase population immunity to serotype 2 transmission or prevent cVDPV2 outbreaks [10]. Thus, after indigenous OPV2-related virus circulation stops following the switch, immunity levels in populations could potentially support the transmission of OPV2-related viruses imported from populations that still use OPV2 (e.g., across shared borders), even with use of IPV in RI. Once these OPV2-related viruses can establish circulation, they could evolve to become cVDPV2s that cause outbreaks requiring aggressive mOPV use in outbreak response and threaten the polio endgame [6, 7].

Given the difficult logistics involved with a tightly-synchronized global switch, we recognize the opportunity to use modeling to explore the extent to which suboptimal synchronization results in potential spread of OPV2-related viruses among countries that switch from tOPV to bOPV at different times. Using the global model, [4] this analysis examines the risks associated with a non-synchronous switch. We focus on characterizing the vulnerability of populations to the circulation of imported OPV2-related viruses and we do not consider the possibility of inadvertent OPV2 use or OPV2 used for outbreak response after the switch [7]. Vulnerability depends primarily on population immunity to transmission [15] and consequently we do not consider in detail the permeability of borders between populations or the consequences of importations that lead to the establishment of ongoing transmission of OPV2-related viruses, which we leave to future studies.

## Methods

The global model [4] integrates a previously developed deterministic, differential equation-based (DEB) poliovirus transmission and OPV evolution model [16, 17] (i.e., the DEB model) with stochastic poliovirus reintroductions after OPV cessation, economic model inputs, characterization of the global variability in conditions that affect poliovirus transmission and vaccination impacts, and a global mixing structure that generates poliovirus exportations to random populations. For this analysis, we use the DEB model and global variability characterization, but we do not consider the economics, stochastic risks, and global mixing structure. Given that the analysis focuses on vulnerability and does not involve the consequences of any random cross-border exportations or other stochastic events, all model results presented in this study remain deterministic (i.e., a single model realization).

Based on an extensive expert review [12, 18, 19] and model calibration process, [16, 17] the DEB model characterizes eight immunity states associated with maternal antibodies, IPV vaccination, and live poliovirus (LPV, i.e., OPV, OPV-related, VDPV, and WPV) infection, five stages of waning of immunity to poliovirus transmission, fecal-oral and oropharyngeal transmission, six infection stages with varying degrees of infectiousness, serotype differences in basic reproduction numbers (R_0_ values, representing measures of inherent transmissibility of polioviruses in a population defined as the average number of secondary infections generated by a typical infection in an entirely susceptible population [20]) and paralysis-to-infection ratios (PIRs), OPV evolution across 20 reversion stages, and poliovirus die-out. The immunity states and multi-stage processes represent conceptual constructs to approximate the evidence about immunity to poliovirus transmission, infection, and OPV evolution [12, 18, 19]. The model produces behavior consistent with the evidence about WPV incidence and die-out as a function of vaccine use, secondary OPV spread and cVDPV emergence or lack thereof, and the age distributions of cases in 10 actual populations encompassing all three serotypes and a wide range of conditions related to poliovirus transmission [14, 16, 17]. The DEB model indigenously tracks OPV viruses (stage 0) introduced by vaccination as they evolve during transmission through 19 subsequent stages with increasing R_0_ values and PIRs as long as low population immunity to transmission permits their prevalence to remain above a certain transmission threshold. OPV-related viruses that make it to the last reversion stage (i.e., stage 19) circulate as fully-reverted VDPVs with the same assumed R_0_ and PIR as homotypic WPVs. Thus, cVDPV emergence within populations occurs deterministically in the model and primarily depends on population immunity to transmission. The DEB model assumes that all immunity states associated with IPV vaccination or LPV infection benefit from permanent protection from paralysis, but that the ability to asymptomatically participate in transmission varies by nature of the immunity (i.e., IPV-only vs. LPV or IPV and LPV) and by waning stage. We express population immunity to transmission as the proportion of the population effectively immune to transmission (EIPM), taking into account age-heterogeneous mixing and the relative contribution to transmission of individuals in different immunity states [21, 22]. The mixing-adjusted net reproduction number (R_n_) represents a closely-related measure and equals the R_0_ of a poliovirus strain (serotype, reversion stage) in a given setting multiplied by one minus the EIPM [22]. R_n_ represents the average number of secondary infections generated by a typical infection, taking into account both the R_0_ of the virus and population immunity to transmission. Given its normalization by the R_0_, a threshold value of 1 applies for R_n_ (i.e., R_n_^*^ = 1), above which poliovirus strains can establish or continue circulation and below which imported or circulating polioviruses will ultimately die out. In contrast, the analogous threshold for EIPM (EIP* = 1–1/R_0_) depends on the R_0_ of the virus strain in a given setting. We take advantage of the comparability of R_n_ values and use them for this analysis as measures of the vulnerability of a population to circulation of different poliovirus strains. However, we emphasize that a specific R_n_ value implies different levels of population immunity to transmission for populations with different R_0_ values. For example, an R_n_ of 1 in a population with poliovirus transmissibility characterized with an R_0_ of 10 corresponds to an EIPM of 0.9 (i.e., 1–1/R_0_), while it corresponds to an EIPM of only 0.8 in a population with poliovirus transmissibility characterized with an R_0_ of 5 (i.e., higher inherent transmissibility necessitates greater immunity to prevent or stop transmission). R_n_ changes over time as a result of seasonality in R_0_ and changes in population immunity due to RI, SIAs, exposure to circulating LPVs, population growth, and waning of immunity to poliovirus transmission.

The global model [4] divides the world into 710 subpopulations of approximately 10 million people (as of 2013) with characteristics selected to represent the global variability in transmissibility (i.e., R_0_ and seasonality, role of oropharyngeal transmission, strength of age-preferential mixing), vaccination program quality (i.e., RI coverage and SIA frequency and quality), and surveillance quality (i.e., number of paralytic cases needed to detect an outbreak). The global model groups the subpopulations into epidemiological blocks consisting of 10 preferentially-mixing subpopulations that share the same World Bank income level, [23] same historical use of polio vaccines, and similar R_0_ values. In the model, the R_0_ values of all serotypes and reversion stages in a given population depend directly on the assumed R_0_ of WPV1, with WPV2 and WPV3 R_0_ values equal to 90 % and 80 % of the WPV1 R_0_ value, respectively. We use the WPV1 R_0_ value to represent the inherent transmissibility of polioviruses and as a proxy for all conditions that affect poliovirus transmissibility in different populations (e.g., hygiene and sanitation, population density, climate) [4, 16, 24]. Based on RI coverage and R_0_ values, the global model assumes simplified SIA schedules for all subpopulations that used OPV only (i.e., no IPV) for RI as of 2013, which includes 520 subpopulations in low- and middle-income blocks. From 2010 through 2014, the first annual SIA in subpopulations that conduct at least 1 per year uses tOPV, while most subsequent annual SIAs use bOPV. From January 1, 2015, the global model assumes that all blocks introduce at least 1 IPV dose into their RI schedule and that the world implements tOPV intensification by using tOPV instead of bOPV in one or two annual SIAs in all subpopulations that conduct three or more annual SIAs (corresponding to populations with less than 90 % RI coverage) to boost population immunity to serotype 2 polioviruses before the switch in April 2016 [4, 5]. Although the switch plan includes a 2-week window between April 17 and May 1, 2016 for the switch, our existing global model dates back from before the specific window became public and assumed that all countries switch exactly on April 1, 2016 [4]. Thus, for consistency with analyses of the existing global model results, we adopt April 1, 2016 instead of the actual 2-week window as the baseline switch date for all populations that switch on time.

We perform three sets of analyses. Analysis I reports the distribution of R_n_ values as a function of time since the switch for different reversion stages of OPV2-related viruses among the 520 subpopulations in the global model that used OPV-only as of 2013. We consider both the base case results with tOPV intensification and an alternative scenario without tOPV intensification that does not replace some bOPV SIAs with tOPV in the run up to the switch [4]. We also consider the relationship between various population-specific model inputs and the time since the switch until the R_n_ of OPV2 exceeds 1, as well as the impact of seasonal fluctuations on the distribution of R_n_ values of OPV2.

Analysis II explores the relationship between the R_n_ at the time of the switch, the time until indigenous OPV2-related viruses die out, and the time until imported OPV2-related viruses in different reversion stages can establish transmission. For this analysis, we use a hypothetical population with setting-specific inputs listed in the top section of Table 1 reflecting properties approximately like northern India [4, 9, 25]. However, to focus the analysis on demonstrating key concepts and control for the effect of seasonality on die-out and R_n_ values, we do not include seasonal variation in R_0_. To attain different R_n_ values at the time of the switch, we vary the switch date to occur at different times after two tOPV SIAs in early 2015. We conduct the analysis for R_0_ values of WPV1 of 10 or 13 to examine the effect of R_0_. As previously noted, the R_0_ of WPV1 serves as a proxy for all conditions that affect transmissibility of all polioviruses in different populations, and the model appropriately uses lower relative R_0_ values for serotype 2.

**Table 1:** Setting-specific model inputs for Analyses II and III, adapted from the global model [4] and adopting all other global model assumptions, including generic inputs from the DEB model.[16, 17]

Region	R_0_	α	pd	κ	tr	POL3	TC	P_rm_	p^oro^	# tOPV SIAs in 2015	Year of IPV introduction	Hypothetical switch date
Analysis II:												
Hypothetical population	10 or 13	0	NA	0.35	0.6	0.3	0.8	0.7	0.3	2	2015	Variable after last SIA in 2015
Analysis III:												
Population like northern India	13	0.2	180	0.35	0.6				0.3	2	2015	
- Pop. A, subpop. 0 (under-vacc.)						0.3	0.8	0.7				Mid-2015
- Pop. A, subpop. 1 (general)						0.6	0.95	0.5				Mid-2015
- Pop. B, subpop. 0 (under-vacc.)						0.3	0.8	0.7				April 1, 2016
- Pop. B, subpop 1 (general)						0.6	0.95	0.5				April 1, 2016
Population like northern Pakistan/Afghanistan	11	0.2	180	0.35	0.65				0.3		2015	
- Pop. A, subpop. 0 (general)						0.6	0.8	0.7		2		Mid-2015
- Pop. A, subpop. 1 (general)						0.6	0.8	0.7		2		Mid-2015
- Pop. B, subpop. 0 (under-vacc.)						0.1	0.35	0.95		3		April 1, 2016
- Pop. B, subpop 1 (general)						0.6	0.8	0.7		2		April 1, 2016
Population like Ukraine	6	0.4	180	0.45	0.74				0.8	0	2005	
- Pop. A, subpop. 0 (general)						0.7^a^	0.8	0.7				Mid-2015
- Pop. A, subpop. 1 (general)						0.7^a^	0.8	0.7				Mid-2015
- Pop. B, subpop. 0 (under-vacc.)						0.3 ^a^	0.8	0.7				April 1, 2016
- Pop. B, subpop 1 (under-vacc. )						0.3^a^	0.8	0.7				April 1, 2016

Model input symbols: [3, 16] *R*_*0*_ average annual basic reproduction number for WPV of serotype 1, *α* seasonal amplitude of R_0_, defined as the “proportional change in R_0_ due to seasonality” [16, p. 717], *pd* peak day of R_0_, *κ* strength of preferential mixing between age groups, defined as the “proportion of contacts reserved for individuals within the same mixing age group”[16, p. 717], *tr* take rate of serotype 2 tOPV, *POL3* RI coverage with 3 or more non-birth doses, *TC* true coverage of each SIA, *P*_*rm*_ repeated missed probability of each SIA, p^oro^, proportion of transmissions via oropharyngeal route

^a^Assume POL3 = 90 % prior to 2010

Analysis III explores the time window of exposure to OPV2-related viruses as a result of a non-synchronous switch in realistic populations taken from the global model. The second section of Table 1 lists the assumed setting-specific model inputs for these populations. Unlike the global model mixing structure with blocks of 10 subpopulations each, for this analysis we consider the simplest case of two populations each consisting of two equally-sized subpopulations, which may include one with characteristics typical of an under-vaccinated subpopulation and one with characteristics typical of the general population [4, 16, 22, 25]. The populations may represent countries or states that potentially switch at different times, and we assume subpopulations of the same population always switch at the same time. We used a two-population, four-subpopulation model because it represents the simplest possible structure to characterize heterogeneity in switch times and population immunity.

Part of analysis III considers two populations with high R_0_ and under-vaccinated subpopulations (i.e., conditions similar to northern India and northern Pakistan and Afghanistan) using assumptions from the global model. The remainder of analysis III specifically models a situation with disrupted immunization prior to the switch (e.g., the Ukraine), for which we assumed several departures from the global model to accommodate a simplified vaccination history. Specifically, in an abbreviated run-up, this Ukraine-like model assumes that RI with OPV-only started in 1980 and eliminated indigenous WPVs, 4 SIAs occurred in the late 1990s, and a switch to an IPV/OPV sequential schedule occurred in 2005 (i.e., 2 doses of IPV followed by 2 doses of tOPV). We further assume that RI coverage with 3 or more poliovirus vaccine doses decreases from 90 % everywhere before 2010 to 30 % in under-vaccinated subpopulations and 70 % in the better-vaccinated general population from then forward, reflecting deteriorating immunization and leading to approximately 50 % national coverage and significant heterogeneity. We considered a hypothetical worst case scenario in which the better-vaccinated half of the population switches prematurely from tOPV to bOPV in mid-2015 while the under-vaccinated subpopulations continue to use tOPV until the global switch in April 2016. An alternative scenario for this population delays the date of IPV introduction to the beginning of 2017, assuming a 3-dose OPV-only RI schedule until then. We did not include the tOPV mop-up activities that apparently controlled the serotype 1 cVDPV transmission responsible for 2 detected Ukrainian polio cases in 2015 [26].

## Results

**Fig. 1:**
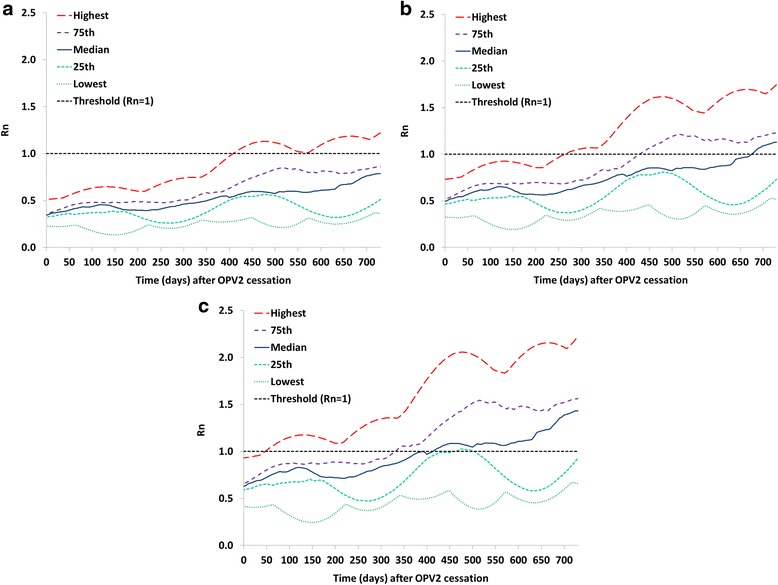
Analysis I results showing selected percentiles from the distribution of net reproduction numbers (R_n_ values) for the 520 subpopulations in the global model [4] that used OPV-only as of 2013 with base case assumptions including tOPV intensification before the tOPV to bOPV switch in April 2016. **a** R_n_ values for OPV2 (stage 0). **b** R_n_ values for stage 10 OPV2-related virus. **c** R_n_ values for VDPV2 (stage 19)

Figure 1 shows the results of Analysis I in the form of selected percentiles from the distribution of R_n_ values for the 520 subpopulations in the global model that used only OPV as of 2013 for OPV2, identical to the parent vaccine strain (stage 0), partially-reverted OPV2-related virus in stage 10, and fully-reverted VDPV2 (stage 19). Fig. 1a suggests that with well-implemented tOPV intensification everywhere ahead of the switch, it takes over a year for the R_n_ of OPV2 to exceed 1 in the first subpopulation, and over 2 years until it exceeds 1 in over 75 % of the subpopulations. Thus, even in the event of relatively large gaps in switch times, populations that switch on time sustain high enough expected population immunity to transmission to prevent established circulation of OPV2 virus imported from populations that continue to use OPV2. However, continued OPV2 use implies the existence of partially-reverted OPV2-related viruses evolved from OPV2 to varying degrees, because even vaccine recipients can excrete partially-reverted OPV2-related viruses. This suggests some potential for OPV2-related viruses in higher reversion stages to circulate in the event of a non-synchronous switch. In our model, some prevalence (i.e., above the transmission threshold) of partially-reverted OPV2-related viruses up to stage 10 typically exists in the context of regular OPV2 use in RI and SIAs that sustain high enough population immunity to serotype 2 to prevent transmission and evolution to VDPV2. Figure 1b shows that it takes well over 6 months after a national switch until the first subpopulation can support transmission of a stage 10 OPV2-related virus, assuming adequate tOPV intensification. Figure 1c shows that fully-reverted VDPV2 viruses can begin to establish transmission as early as 50 days after the switch in some subpopulations, which illustrates the importance of stopping all persistent cVDPV2s prior to OPV2 cessation [27]. Thus, in the event of indigenous cVDPV2 circulation in a population that switches later, exported cVDPV2 outbreak viruses may lead to established circulation in other populations for differences in switch times as short as 2 months.

**Fig. 2:**
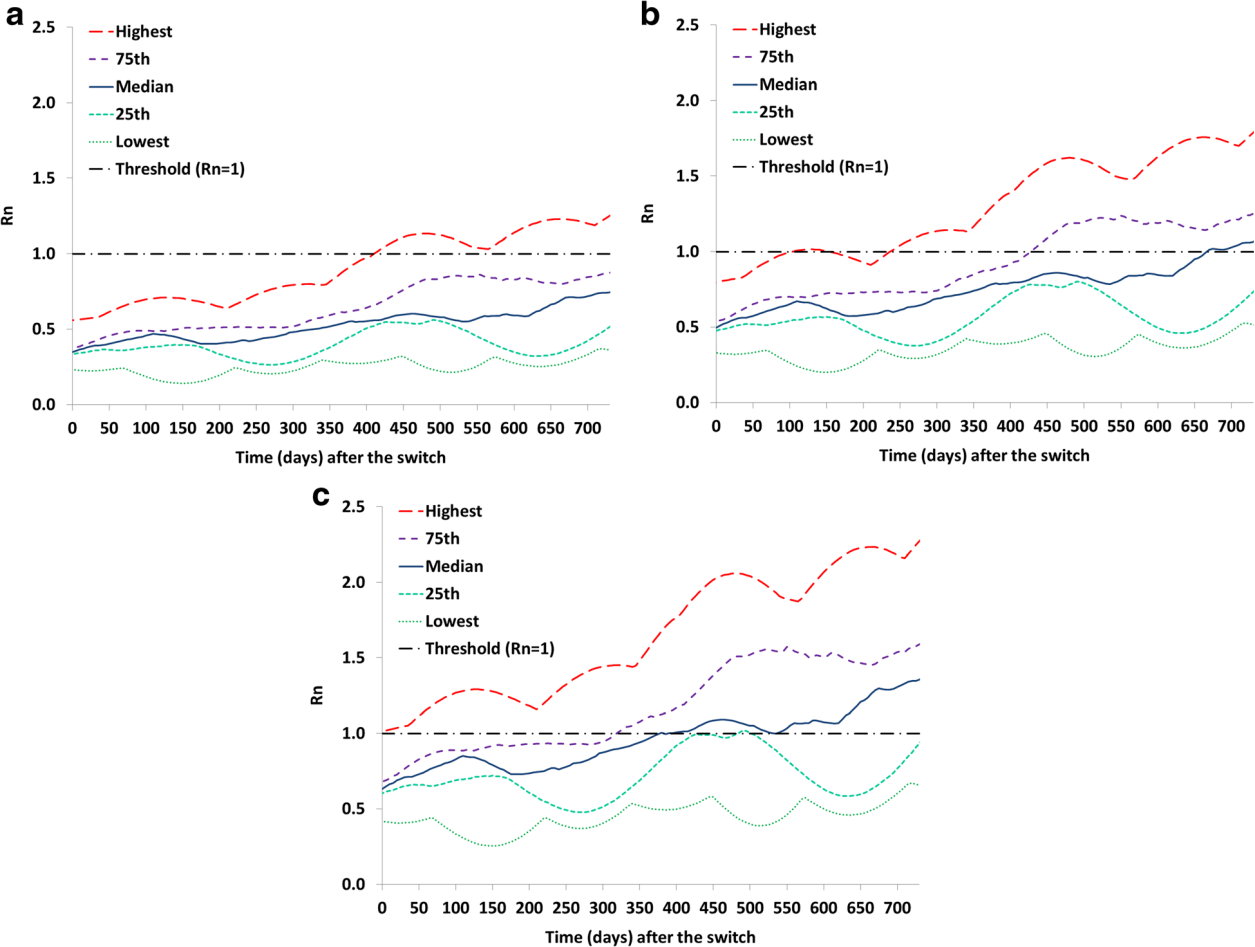
Analysis I results showing selected percentiles from the distribution of net reproduction numbers (R_n_ values) for the 520 subpopulations in the global model [4] that used OPV-only as of 2013 assuming no tOPV intensification before the tOPV to bOPV switch in April 2016. **a** R_n_ values for OPV2 (stage 0). **b** R_n_ values for stage 10 OPV2-related virus. **c** R_n_ values for VDPV2 (stage 19)

Figure 2 shows a variation of Analysis I without tOPV intensification but all else equal. Failure to intensify tOPV use in a population reduces the time until OPV2-related viruses can establish circulation and thus increases the vulnerability of that population to importation of an OPV2-related virus from a population still using tOPV in a non-synchronized switch. For example, the time since the switch until the R_n_ for stage 10 OPV2-related viruses exceeds 1 in at least one subpopulation decreases from over 6 months with tOPV intensification to less than 100 days without tOPV intensification. With respect to VDPV2s, the R_n_ already exceeds 1 at the time of the switch in one subpopulation without tOPV intensification, which results in an indigenous cVDPV2 outbreak in this subpopulation following the switch [4, 7].

Table 2 explores the relationship between population-specific properties and the time until the R_n_ of OPV2 (stage 0) virus exceeds 1 for the 520 subpopulations characterized in Fig. 1 (i.e., base case with tOPV intensification). The second column of Table 2 reports the number of subpopulations for which the time until the R_n_ of OPV2 (stage 0) virus exceeds 1 falls into the ranges shown in the first column. The R_n_ prior to the switch (third column) indicates the starting point of vulnerability, averaged over a full year before the switch to control for seasonality, after which the removal of OPV2 from all immunization activities leads to increased vulnerability to any introduction of OPV2 (stage 0) virus. Higher R_n_ before the switch generally leads to shorter times until R_n_ of OPV2 (stage 0) virus exceeds 1, although the relationship is imperfect due to other factors that also influence the time until R_n_ of OPV2 (stage 0) virus exceeds 1 (e.g., RI coverage with IPV, the relative contribution of oropharyngeal transmission (p^oro^), seasonality in R_0_). Table 2 shows that subpopulations with high R_0_ values and little contribution of oropharyngeal transmission tend to reach an R_n_ for OPV2 (stage 0) viruses of 1 the soonest. The global model assumes that SIA frequency inversely relates to RI coverage so that SIAs can close immunity gaps in areas with poor RI coverage. Consequently, different combinations of RI coverage values and numbers of tOPV SIAs can produce similar times until OPV2 exceeds 1. Although subpopulations with R_0_ values below 9 typically sustain an R_n_ of OPV2 (stage 0) virus below 1 for many years, Table 2 shows two exceptions (i.e., a chronically under-vaccinated subpopulation with an R_0_ of 8 that reaches this point within approximately 2 years and a sub-optimally vaccinated subpopulation with an R_0_ of 7 and very strong seasonality that reaches this point during high seasons within 3 years).

**Table 2:** Relationship between the time after the switch until the net reproduction number (R_n_) of OPV2 (stage 0) virus exceeds 1, the R_n_ of OPV2 (stage 0) virus at the time of the switch, and population-specific properties based on the populations from the global model [4] represented in the distributions of Fig. 1 (analysis I base case with tOPV intensification)

Time (years) since switch until R_n_ of OPV2 > 1	# subpopulations in time range (out of 520 with OPV-only)	Properties of subpopulations in range						
		Average R_n_ before switch^a^	R_0_^b^	p^oro^	tr	POL3	# tOPV SIA during intensification (2015–2016)	TC
1.5–1.74	16	0.37–0.44	11–13	0.3	0.6–0.7	0.3	6	0.5
						0.6	6	0.8
						0.9	2	0.8
1.75–1.99	65	0.36–0.51	10–13	0.3	0.6–0.7	0.1	7	0.35
						0.3	6	0.8
						0.6	4	0.5
						0.6	6	0.8
						0.98	2	0.95
2–2.24	100	0.36–0.42	8	0.3	0.7	0.05	7	0.15
			10–11	0.3	0.65–0.7	0.3	6	0.8
						0.6	4	0.5–0.8
						0.6	6	0.5
						0.9	2	0.8–0.95
2.25–2.49	20	0.37	10	0.3	0.7	0.6	4	0.95
						0.9	2	0.95
2.5–2.74	20	0.33–0.35	9	0.3	0.65–0.7	0.6	4	0.8
						0.9	2	0.8
						0.98	0	0.95
2.75–3	30	0.25–0.36	7–9	0.3	0.7	0.3	6	0.5
						0.6	4	0.8
						0.9	2	0.8
				0.8	0.65	0.6	4	0.95
3–3.99	58	0.25–0.36	7–8	0.3	0.7–0.72	0.3	6	0.8
						0.6	4	0.5–0.8
						0.9	2	0.8
						0.98	0	0.95
				0.5	0.72	0.6	4	0.8
				0.8	0.65	0.9	2	0.95
						0.98	0	0.95
4–4.99	2	0.29	7	0.5	0.72–0.73	0.6	4	0.8
						0.9	2	0.8
5–7.49	30	0.27–0.35	6–8	0.5–0.6	0.73	0.6	4	0.8
						0.9	2	0.8–0.95
7.5–9.99	3	0.30	7	0.6	0.73	0.6	4	0.8
10–14.99	16	0.31–0.36	7–8	0.6	0.73–0.74	0.6	4	0.8
						0.9	2	0.8–95
						0.98	0	0.95
15–19.99	116	0.3–0.37	7–8	0.6	0.73–0.74	0.9	2	0.8
						0.98	0	0.95
>20	44	0.3–0.35	6–7	0.6–0.8	0.73–0.75	0.9	2	0.95
						0.98	0	0.95

Model input symbols: [3, 16] *R*_*0*_ average annual basic reproduction number for WPV of serotype 1, *tr* take rate of serotype 2 tOPV, *POL3* RI coverage with 3 or more non-birth doses, *TC* true coverage of each SIA, *p* ^*oro*^ proportion of transmissions via oropharyngeal route

^a^Defined as the average R_n_ of OPV2 over the one-year period preceding the switch

^b^The global model uses R_0_ for WPV1 to characterize variability in subpopulations, R_0_ for serotype 2 WPV equals 0.9 times the values shown in this column

Table 3 shows the relationship between population-specific properties and the time until the R_n_ of OPV2 (stage 0) virus exceeds 1 for the 520 subpopulations characterized in Fig. 2 (i.e., similar to Table 2 except without tOPV intensification). In general, without tOPV intensification the time until R_n_ of OPV2 (stage 0) virus exceeds 1 decreases (i.e., shorter time, increased risk) for subpopulations affected by the tOPV intensification policy (i.e., those with higher R_0_ values and low RI coverage), except for the population in which an indigenous cVDPV2 emerges because of the failure to intensify tOPV use, which leads to subsequent higher immunity due to the outbreak and response. Comparison of Tables 2 and 3 shows that tOPV intensification did not affect subpopulations with relatively lower R_0_ values and higher RI coverage that already only conduct tOPV SIAs or conduct no SIAs.

**Table 3:** Relationship between the time after the switch until the net reproduction number (R_n_) of OPV2 (stage 0) virus exceeds 1, the R_n_ of OPV2 (stage 0) virus at the time of the switch, and population-specific properties based on the populations from the global model [4] represented in the distributions of Fig. 2 (analysis I without tOPV intensification)

Time (years) since switch until R_n_ of OPV2 > 1	# subpopulations in time range (out of 520 with OPV-only)	Properties of subpopulations in range						
		Average R_n_ before switch^a^	R_0_^b^	p^oro^	tr	POL3	# tOPV SIA during 2015–2016	TC
1.0–1.45	40	0.41–0.49	12–13	0.3	0.6	0.3	4	0.35
						0.6	4	0.8
						0.6	4	0.95
						0.9	2	0.8
						0.98	2	0.95
1.5–1.74	10	0.37–0.44	11	0.3	0.7	0.3	4	0.5
						0.6	4	0.8
						0.9	2	0.8
						0.98	2	0.95
1.75–1.99	30	0.55	8	0.3	0.7	0.05	4	0.15
		0.35	9	0.3	0.7	0.6	2	0.8
		0.35–0.43	9–12	0.3	0.65–0.7	0.3	4	0.5
						0.6	4	0.5–0.95
						0.9	2	0.8–0.95
2–2.24	103	0.36–0.41	10–11	0.3	0.65–0.7	0.3	4	0.8
						0.6	4	0.8–0.95
						0.9	2	0.8–0.95
						0.98	0	0.8
2.25–2.49	10	0.37	10	0.3	0.7	0.9	2	0.95
2.5–2.74	18	0.33–0.38	9	0.3	0.65–0.7	0.9	2	0.8
						0.98	0	0.95
2.75–3	31	0.26–0.37	7–9	0.3	0.7–0.72	0.3	4	0.5
						0.6	2	0.8
						0.9	2	0.8
						0.98	0	0.8
				0.8	0.65	0.6	2	0.95
3–3.99	67	0.25–0.39	7–8	0.3	0.7–0.72	0.3	4	0.8
						0.6	2	0.5–0.8
						0.9	2	0.8
						0.98	0	0.95
				0.5	0.72	0.6	2	0.8
				0.8		0.9	2	0.95
						0.98	0	0.95
		0.38^c^	11	0.3	0.65	0.6	4	0.8
		0.53^c^	11	0.3	0.65	0.1	4	0.35
4–4.99	2	0.29	7	0.5	0.72–0.73	0.6	2	0.8
						0.9	2	0.8
5–7.49	30	0.27–0.35	6–8	0.5–0.6	0.73	0.6	2	0.8
						0.9	2	0.8–0.95
7.5–9.99	3	0.30	7	0.6	0.73	0.6	2	0.8
10–14.99	16	0.31–0.36	7–8	0.6	0.73–0.74	0.6	2	0.8
						0.9	2	0.8–95
						0.98	0	0.95
15–19.99	116	0.3–0.37	7–8	0.6	0.73–0.74	0.9	2	0.8
						0.98	0	0.95
>20	44	0.3–0.35	6–7	0.6–0.8	0.73–0.75	0.9	2	0.95
						0.98	0	0.95

Model input symbols: [3, 16] *R*_*0*_ average annual basic reproduction number for WPV of serotype 1, *tr* take rate of serotype 2 tOPV, *POL3* RI coverage with 3 or more non-birth doses, *TC* true coverage of each SIA, *p* ^*oro*^ proportion of transmissions via oropharyngeal route

^a^Defined as the average R_n_ of OPV2 over the one-year period preceding the switch

^b^The global model uses R_0_ for WPV1 to characterize variability in subpopulations, R_0_ for serotype 2 WPV equals 0.9 times the values shown in this column

^c^Population shows long time until R_n_ of OPV2 exceeds one because of an indigenous cVDPV2 outbreak in one of its subpopulations following the switch and a subsequent mOPV2 response

The results in Figs. 1 and 2 suggest that the risk of cVDPV2 outbreaks associated with a non-synchronous switch will depend on the size of the gap in switch times because populations will become increasingly vulnerable to transmission of OPV2-related viruses after the switch. Given that after the switch RI doses of IPV, which provides only limited intestinal immunity, [10–12] will represent the only poliovirus vaccine available for RI for serotype 2, the tOPV-induced population immunity to serotype 2 transmission at the time of switch significantly affects the time it takes until imported OPV2-related viruses from subpopulations that still use OPV2 can establish transmission.

**Fig. 3:**
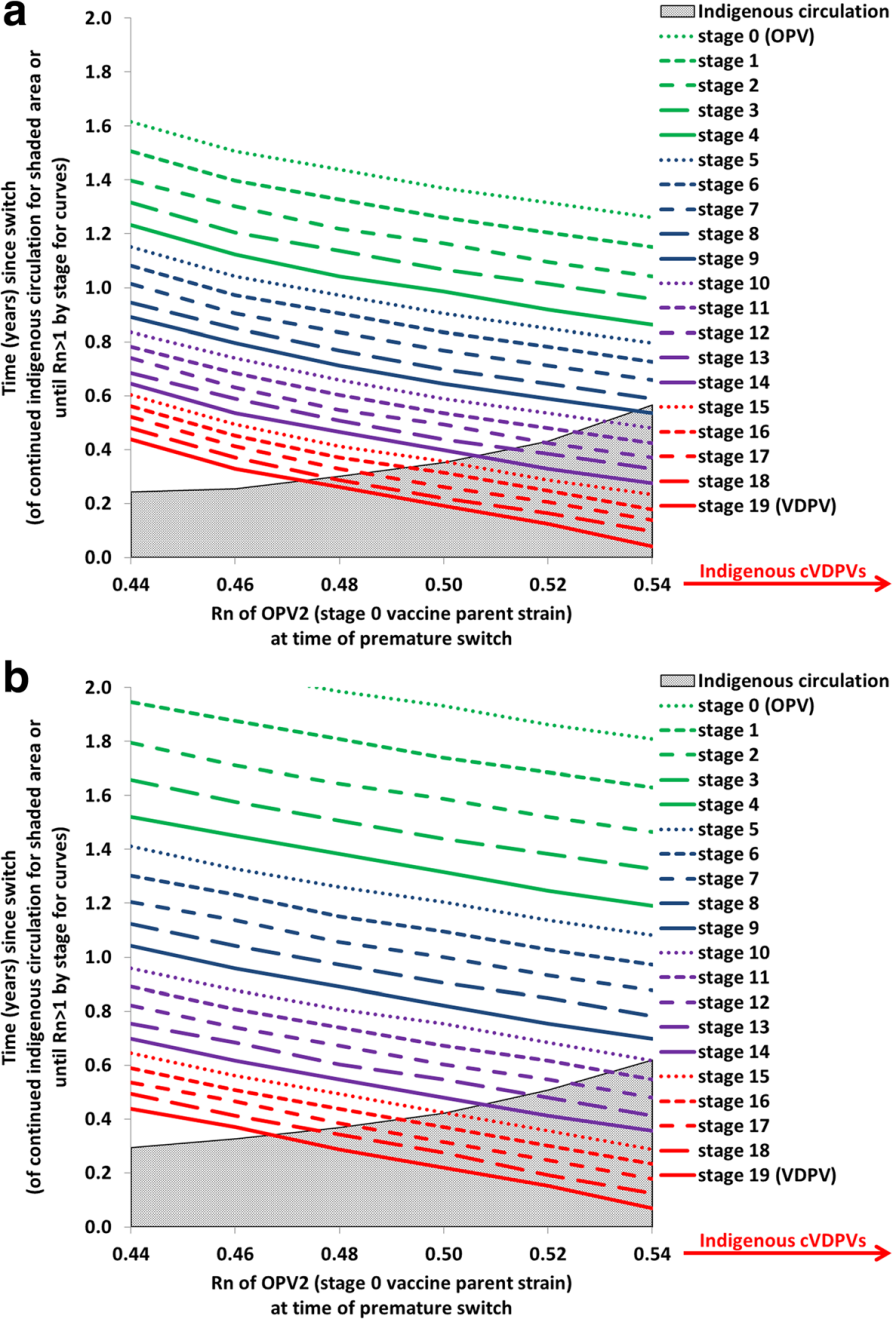
Analysis II results showing the relationship between net reproduction number (R_n_) at the time of the switch and time until OPV2-related viruses in different reversion stages can establish circulation (i.e., time until R_n_ becomes greater than 1) in a hypothetical population (see Table 1) The shaded areas show the duration of indigenous circulation of OPV2-related viruses following the switch, which continues indefinitely if cVDPV2s emerge indigenously in the absence of an outbreak response. **a** Baseline R_0_ for WPV1 equals 13. **b** Baseline R_0_ for WPV1 equals 10

Analysis II further illustrates the importance of the tOPV-induced population immunity to serotype 2 transmission at the time of the switch by showing in Fig. 3 the time until OPV2-related viruses in different reversion stages reach an R_n_ exceeding 1 as a function of the R_n_ of OPV2 (stage 0) at the time of the switch in a hypothetical population with properties provided in Table 1. The two panels of Fig. 3 show the results with different inherent R_0_ values (expressed as R_0_ values for WPV1) but all else equal. In the shaded areas, Fig. 3 also shows the relationship between the R_n_ of OPV2 at the time of the switch and the amount of time during which indigenous OPV2-related viruses still exist and presumably represent the primary source of national risk. The R_n_ values at the time of the switch on the x-axis start at 0.44 because for the modeled hypothetical population with a baseline R_0_ of 13, this represents the lowest R_n_ value attainable by tOPV intensification (i.e., shortly after the last 2 tOPV SIAs). After stopping the use of tOPV, OPV2-related viruses remain present in the population for some time because it takes time until tOPV recipients stop excreting OPV2-related viruses, and some secondary infections with OPV2-related viruses may occur as long as the prevalence of at least one reversion stage remains above the transmission threshold. At the lowest attainable R_n_ (0.44), indigenous OPV2-related viruses die out rapidly within 3 months (Fig. 3a), after which exposure to any OPV2-related viruses from populations that did not yet switch represents the only switch-related risk. Because of the low R_n_ at the time of the switch (0.44), it takes approximately 5 months until imported VDPV2s can establish circulation and approximately 10 months until stage 10 viruses can establish circulation in this hypothetical population.

As R_n_ at the time of the switch increases, the time until any imported OPV2-related viruses can establish circulation decreases, but indigenous circulation also continues for longer because higher R_n_ values mean that each OPV2-related virus infection generates more new infections. Thus, in the shaded area before die-out of indigenous OPV2-related viruses, importations due to a non-synchronous switch present a threat that does not already exist indigenously only if the populations that continue OPV2 use for longer than the modeled population export more reverted viruses than the indigenous viruses. For example, for an R_n_ of 0.54 at the time of the switch, indigenous circulation of OPV2-related viruses, which involves reversion stages 0 through 8 (not shown), continues for 6 months, during which time only imported viruses in stage 9 or higher could establish circulation and pose a larger threat than the indigenous viruses (Fig. 3a). After indigenous circulation stops, lower-stage OPV2-related viruses could establish circulation, but importation of these viruses requires a relatively long gap in switch times (i.e., between countries that switch at different times). For values of R_n_ for OPV2 (stage 0) at the time of the switch over 0.54, the higher R_n_ values for OPV2-related viruses at higher reversion stages lead to higher prevalence values of more reverted OPV2-related viruses that continue to evolve to even higher reversion stages, allowing a virus to appear at a reversion stage for which R_n_ > 1 before all OPV2-related viruses die out. In the absence of vaccination with tOPV to sustain population immunity, this results in an indigenous cVDPV2 outbreak in this modeled population, which makes the risks associated with non-synchronous cessation a secondary concern.

Figure 3b shows the impact of changing the baseline R_0_ for WPV1 from 13 to 10. As we lower R_0_, similar R_n_ values as in Fig. 3a occur for lower population immunity to transmission, and thus the x-axis ranges in Figs. 3a and b represent different levels of population immunity to transmission at the time of the switch. Figure 3b shows that indigenous cVDPV2s still emerge above the same threshold R_n_ at the time of the switch of approximately 0.54. Changing the baseline R_0_ from 13 to 10 increases the time until any imported OPV2-related viruses can establish transmission, which suggests that for lower R_0_ values, relatively longer gaps in switch times can occur without a significant risk of established circulation from imported OPV2-related viruses.

**Fig. 4:**
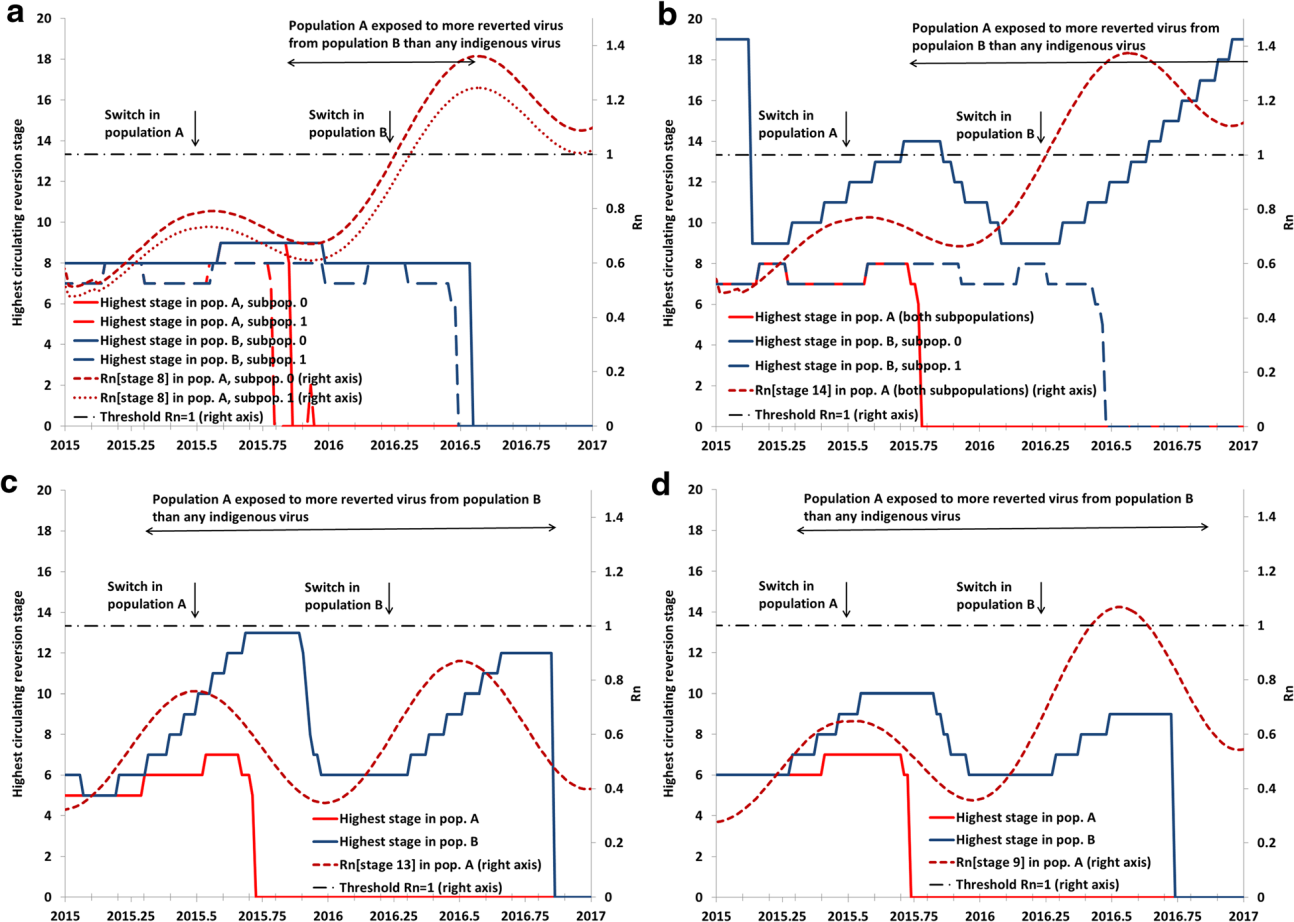
Analysis III results showing examples of non-synchronous switch dynamics in various realistic settings in a two-population, four-subpopulation model. **a** Setting like northern India. **b** Setting like northern Pakistan and Afghanistan. **c** Setting like Ukraine, assuming IPV use since 2005. **d** Setting like Ukraine, assuming no IPV use until 2017

While Fig. 3 focuses on the relationship between the R_n_ at the time of the switch in the potentially importing populations (i.e., those that switch earlier), it also reveals the influence of the mix of OPV2-related viruses in the exporting populations (i.e., those that switch later), which depends on their population immunity to serotype 2 transmission. Analysis III examines the interplay between importing and exporting populations using two-population and four-subpopulation models with realistic properties (Fig. 4). All panels of Fig. 4 show the highest reversion stage of OPV2-related virus circulating in each subpopulation (left axis), as well as the R_n_ values for the highest reversion stage of OPV2-related viruses that the population that switches early (i.e., population A) gets exposed to from the population that switches late (i.e., population B). The highest reversion stage that circulates in a population depends on the rate of introduction of OPV2 viruses through tOPV vaccination and the level of population immunity to transmission. Even with high population immunity and R_n_ of OPV2 (stage 0) well below 1, tOPV use during RI and SIAs implies some prevalence of OPV2 virus above the transmission threshold, which leads to limited transmission (i.e., less than 1 infection per new infection on average) and some evolution to subsequent reversion stages. Thus, with ongoing tOPV use, higher reversion stages can exist at some level in the model even if they do not lead to amplified transmission and cVDPV2 emergence, which only occurs for higher R_n_ values.

Figure 4a models two populations with properties like northern India, [25] which both include under-vaccinated subpopulations with sub-optimal RI and SIA quality. The premature switch in population A approximately 270 days before population B (Table 1) leads to a similar window of approximately 250 days during which partially-reverted viruses exist in population B that no longer circulate in population A. However, of these 250 days, the R_n_ for those viruses exceeds 1 only during the last 100 days, and their prevalence in population B remains too low to trigger any exportations to population A with the global model assumptions for inter-population mixing [4].

In Fig. 4b, we modeled the possibility of exposure to more reverted viruses in a setting like northern Pakistan and Afghanistan (Table 1) by assuming that the under-vaccinated subpopulation in population B continues to use OPV2 after a premature switch in population A. As shown in Fig. 4a, OPV2-related viruses in elevated stages of reversion exist in the under-vaccinated subpopulation of population B and present a risk of exportation to population A. However, because the very low population immunity to serotype 2 transmission in the under-vaccinated subpopulation of population B permits circulation of its own highly-reverted OPV2-related viruses even before the switch in population B, an indigenous cVDPV2 emerges after the switch in the under-vaccinated subpopulation of population B. Thus, it appears that for a gap in switch times of less than a year, an extended period of potential importation of OPV2-related viruses at a high enough reversion stage to establish transmission only occurs if a high potential of indigenous cVDPV2s already exists in the exporting population.

Figure 4c models the case of a population with sub-optimal RI coverage that did not conduct tOPV SIAs for many years, as occurred in the Ukraine. For this modeled temperate setting, we assume much more seasonal variation in R_0_ values, which coupled with low RI in population A (Table 1) results in circulation of OPV2-related viruses at relatively high reversion stages during the high season. Figure 4c shows a very long window of approximately 1.5 years during which the better-vaccinated subpopulations could import more reverted viruses from the under-vaccinated subpopulations than any of its indigenous viruses. This window starts before the premature switch in population A and continues through the switch in population B and subsequent long period of indigenous OPV2-related virus circulation in population B. However, due to its relatively better RI coverage, which includes two IPV doses after the switch in a setting with relatively little fecal-oral transmission, population A sustains high enough population immunity to transmission to prevent established circulation of any of the OPV2-related virus strains circulating in population B. Additional analyses showed that as we lower RI coverage in population B while increasing coverage in population A to fix the national coverage at 50 %, we reach the point at which indigenous cVDPV2s emerge after the switch in population B well before the point at which population A can establish circulation of any imported cVDPV2s from population A. If we decrease coverage in population A while still fixing national coverage at 50 %, then population A becomes more vulnerable to highly-reverted OPV2-related viruses, but population B no longer creates these viruses. As in the other settings, it appears that for a gap in switch times below a year, high vulnerability to imported OPV2-related viruses only occurs when the potential for indigenous cVDPV2s is already very high in the importing and/or exporting population. Figure 4d shows that with a (3-dose) OPV-only schedule instead of the sequential IPV/OPV schedule since 2005, population immunity to transmission remains somewhat higher before the switch, leading to circulation of less-reverted OPV2-related viruses (i.e., highest reversion stage reached of 10 instead of 13). However, after the switch, the absence of IPV RI results in a more rapid decrease in population immunity to transmission, higher R_n_ values for OPV2-related viruses, and a brief period of vulnerability of population A to OPV2-related viruses still circulating in population B.

## Discussion

This study expands on prior game theory arguments for coordinated OPV cessation [27, 28] and demonstrates increased vulnerability of different populations to importation of OPV2-related polioviruses in the event of a non-synchronous switch from tOPV to bOPV. Assuming well-implemented tOPV intensification and, less importantly, use of at least a single dose of IPV in RI programs everywhere, the results overall suggest a time window of approximately 6 months or more after the switch during which residual tOPV-induced population immunity to transmission will prevent imported OPV2-related polioviruses that typically circulate in countries that still use OPV2 [19, 29–31] from establishing circulation. This finite window provides some reassurance that the planned risk management strategies will reduce vulnerability associated with unexpected delays, but overall the analysis confirms the need to globally-coordinate OPV cessation of any serotype, [27, 28] as planned [1, 2, 32]. However, a premature switch in a population with already sub-optimal population immunity to transmission will shorten this finite window. A delayed switch in a population with sub-optimal population immunity to transmission also increases risk because it permits indigenous circulation of more reverted OPV2-related viruses that could export to populations that already switched. Thus, failure to intensify tOPV use prior to the switch not only increases the risk of the creation of indigenous cVDPV2s, [3, 4, 7, 10, 22, 25] but also increases the risk of cVDPV2 outbreaks associated with a non-synchronous switch. To minimize the risk of cVDPV2 outbreaks after the switch, this analysis implies that, regardless of IPV use, all countries should switch as close as possible to the agreed upon global switch date and continue or intensify efforts to maintain high population immunity using tOPV up until the switch. The possibility of a cVDPV2 outbreak due to a failure to fully synchronize the switch, a failure to eliminate existing cVDPV2s through intense tOPV use prior to OPV cessation, [3] or inadvertent tOPV use after the switch [33] reinforces the need for outbreak response preparedness and the stockpiling of mOPV2 and any IPV needed for potential outbreak responses [6, 7].

Our analysis focused on the vulnerability of populations to imported OPV2-related viruses, which countries can directly control with their immunization choices [15]. The degree of cross-border transmission between any populations that might not switch on the same date remains uncertain and more difficult to control. The rate of exportations between communities that share a physical border may exceed the average values for inter-population exportations assumed in the global model [4] and requires further research. In addition, we emphasize that actual importations represent stochastic events, and rare events sometimes occur. Given vulnerability to imported OPV-related viruses, the risks of cross-border transmission will increase with the size of the populations that fail to synchronize OPV cessation. While population immunity to transmission after the switch depends primarily on population immunity to transmission at the time of the switch, IPV use during RI will reduce the rate of decrease in population immunity to transmission, particularly in settings with better hygiene and a lower contribution of fecal-oral versus oropharyngeal transmission, but we emphasize that these populations already represent relatively lower risk populations.

All limitations from the global model [4] and the DEB model [16, 17] carry over to this analysis. Uncertainty about many model inputs may impact our results such as the assumed shape of the waning curve, which affects how fast population immunity to transmission decreases after the switch. The DEB model also does not model the degree of reversion of OPV-related viruses at the individual level, which includes a small fraction (i.e., mathematically, a distributional tail) of healthy vaccine recipients that excrete VDPVs according to the virological definition [34]. While the epidemiological significance of these viruses remains unknown, in our model the average time to evolve from OPV2 to fully-reverted VDPV2s with R_0_ values equal to WPV2 equals approximately 15 times the average individual excretion period [17]. If viruses excreted by some fraction of healthy vaccine recipients represent true VDPVs, then this increases both the indigenous cVDPV2 risk after the switch and the risk of cVDPV2 associated with a non-synchronous switch. This analysis also did not consider the risk of accidental use of tOPV after the switch, which we considered in our global model only for the first year after a globally-coordinated switch [4] and subsequently considered in more detail for specific populations [33]. Any later introductions of OPV2 could establish circulation more easily, and thus successful withdrawal of tOPV from the field and containment from laboratories remain critical [33].

Despite these limitations, our work provides support for efforts to manage the risks associated with the switch including plans to tightly coordinate the switch across all countries and the need to ensure sufficient tOPV supply and use up until the global switch.

## Conclusions

Short national discrepancies in the timing of the tOPV to bOPV switch will likely not significantly increase cVDPV risks due to the insurance provided by tOPV intensification efforts conducted prior to the switch, although countries should all strive to coordinate their national switch within the globally agreed April 17-May 1, 2016 time window to minimize the risks associated with cross-border importations, even in the context of current limitations in IPV supply.

## Abbreviations

bOPV: bivalent oral poliovirus vaccine
cVDPV(2): circulating VDPV (serotype 2)
DEB: differential equation-based
EIP*: threshold effective immune proportion
EIPM: mixing-adjusted effective immune proportion above which infections eventually die out
GPEI: Global Polio Eradication Initiative
IPV: inactivated poliovirus vaccine
iVDPV: immunodeficiency-associated VDPV
LPV: live poliovirus
mOPV: monovalent oral poliovirus vaccine
OPV: oral poliovirus vaccine
OPV13: serotype 1- and 3-containing OPV
OPV2: serotype 2-containing OPV
PIR: paralysis-to-infection ratio
R_0_: basic reproduction number
RI: routine immunization
R_n_: mixing-adjusted net reproduction number
SIA: supplemental immunization activity
tOPV: trivalent oral poliovirus vaccine
VDPV: vaccine-derived poliovirus
WPV(1,2,3): wild poliovirus (serotype 1, 2, or 3, respectively)
